# Phased implementation of spaced clinic visits for stable HIV-positive patients in Rwanda to support Treat All

**DOI:** 10.7448/IAS.20.5.21635

**Published:** 2017-07-21

**Authors:** Sabin Nsanzimana, Eric Remera, Muhayimpundu Ribakare, Tracy Burns, Sibongile Dludlu, Edward J Mills, Jeanine Condo, Heiner C Bucher, Nathan Ford

**Affiliations:** ^a^ Institute of HIV Disease Prevention and Control, Rwanda Biomedical Center, Kigali, Rwanda; ^b^ Basel Clinical Epidemiology and Biostatistics, University Hospital Basel, Basel, Switzerland; ^c^ Swiss Tropical and Public Health Institute, University of Basel, Switzerland; ^d^ UNAIDS, Country office, Kigali, Rwanda; ^e^ Precision Global Health, Vancouver, Canada; ^f^ U.S. President’s Emergency Plan for AIDS Relief, US Embassy, Kigali, Rwanda; ^g^ Department HIV and Global Hepatitis Programme, World Health Organization, Geneva, Switzerland

**Keywords:** antiretroviral therapy, differentiated care, Rwanda, stable patients, Treat All

## Abstract

**Introduction**: In 2016, Rwanda implemented “Treat All,” requiring the national HIV programme to increase antiretroviral (ART) treatment coverage to all people living with HIV. Approximately half of the 164,262 patients on ART have been on treatment for more than five years, and long-term retention of patients in care is an increasing concern. To address these challenges, the Ministry of Health has introduced a differentiated service delivery approach to reduce the frequency of clinical visits and medication dispensing for eligible patients. This article draws on key policy documents and the views of technical experts involved in policy development to describe the process of implementation of differentiated service delivery in Rwanda.

**Discussion**: Implementation of differentiated service delivery followed a phased approach to ensure that all steps are clearly defined and agreed by all partners. Key steps included: definition of scope, including defining which patients were eligible for transition to the new model; definition of the key model components; preparation for patient enrolment; considerations for special patient groups; engagement of implementing partners; securing political and financial support; forecasting drug supply; revision, dissemination and implementation of ART guidelines; and monitoring and evaluation.

**Conclusions**: Based on the outcomes of the evaluation of the new service delivery model, the Ministry of Health will review and strategically reduce costs to the national HIV program and to the patient by exploring and implementing adjustments to the service delivery model.

## Introduction

Sub-Saharan Africa carries the highest burden of HIV, with approximately 70% of all people living with HIV worldwide living in the region. Despite major progress by governments, donors, and international and national implementing partners, access to antiretroviral therapy (ART) in sub-Saharan Africa remains below the global average, with less than 50% of people living with HIV on ART, and coverage is considerably lower in some countries in the region [1].

Rwanda is one of a few countries in sub-Saharan Africa that has achieved high rates of HIV diagnosis and ART coverage, along with high rates of retention and medication adherence with viral suppression [2,3]. As of the end of June 2016, 164,262 people – 78% of all PLHIV in Rwanda – were receiving antiretroviral therapy. Retention in care is high, at 93% after 12 months on treatment, and viral suppression among those on ART and receiving a viral load is also high, at 86% at 12 months and 91.5% and 36 months post-ART initiation [4]. This progress is particularly remarkable given the backdrop of the 1994 genocide which left the health system in disrepair. In recent years, increased ART coverage has evolved with treatment guidelines recommending earlier initiation of ART [5].

The successes of Rwanda’s national HIV programme are closely tied to a series of health system improvements made over the past 15 years, including a strengthened infrastructure, introduction of community-based health insurance, improved workforce skills, and training of community health workers to deliver preventive, diagnostic and therapeutic services at the village level [5–7].

Despite successes, Rwanda faces new challenges and opportunities for increasing and sustaining access to antiretroviral therapy. In July 2016 new guidelines were launched implementing “Treat All,” under which all identified people living with HIV (PLHIV) would immediately begin ART irrespective of immunological or clinical status [6]. This new policy is expected to lead to major benefits in terms of reduced mortality, morbidity and new infections [7–9], but challenges remain to national programme to significantly increase the number of PLHIV receiving ART: there are more than 17,000 individuals who were in pre-ART care by June 2016. An estimated 10% increase in the number of PLHIV needing treatment in Rwanda following the shift to Treat All will add additional PLHIV who need continuous access to ART; With an annual HIV incidence of 0.27% (95% confidence interval: 0.18–0.36%), additional HIV positive individuals are anticipated to enter care each year [10].

The national ART coverage target to reach 81% (the second “90” of the UNAIDS 90–90-90 targets) [11] of PLHIV on ART is expected to be achieved by the end of June 2017, with an estimated 92% of PLHIV enrolled in care and 82% on ART. Nevertheless, approximately half of the 164,262 patients on ART have been on treatment for more than five years, and long-term retention of patients in care is increasingly a major challenge for the national programme.

These two challenges of increasing enrolment and ensuring long-term retention have pushed the Ministry of Health of Rwanda to implement new operational strategies. These include reorganizing the existing patient flow and the frequency of clinical visits and the collection of medication from the pharmacy.

Patient flow has been simplified by removing two steps – registration and vital signs regular checks at HIV clinic reception, clinic consultation for file records; the pharmacist at each clinic has been trained to capture key information required before delivering ARVs at each pick up visit in a short time; and unnecessary visits have been removed such pre treatment and regular counselling sessions.

Previous ART guidelines recommended clinic visits every three months for medical consultations, monthly medication pick up, monthly adherence counselling, and every three months visit for psychosocial support. The latest ART guidelines, issued in July 2016, recommend differentiating ART delivery, with spaced clinic visits and pharmacy pick up for patients who are stable on ART, with the anticipated outcomes of increasing efficiencies at the site level for the benefit of patients and clinical staff, leading to sustainable cost savings.

This article summarizes the sequential policy actions taken towards phased implementation of spaced clinic visits for stable patients in Rwanda.

## Discussion

**Figure 1.**
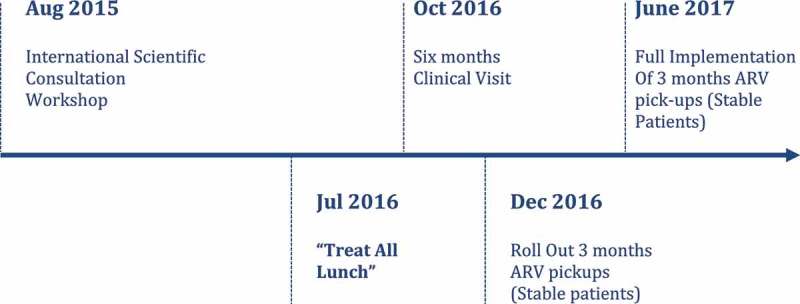
Reconfiguring service delivery to reach “the Second 90.”

Implementation of spaced clinic visits, as outlined in Figure 1 and detailed later has followed a phased approach to ensure that the key steps were defined and agreed by all partners.

### Definition of scope

The first step was to convene a national technical consultation, which was held in August 2015. This immediately proceeded the meeting of the WHO guidelines development group in which the evidence supporting spaced clinic visits was presented [8]. The national technical consultation included representatives from the WHO, UNAIDS, researchers from the University of Rwanda, School of public health, international researchers, members of the national HIV technical working group, the umbrellas of civil society organizations in the fight against HIV and health promotion, the network of people living with HIV, private and public health facilities under the leadership of the HIV control program of the Rwanda Biomedical Centre (RBC), an implementing body of the Ministry of Health. At this August 2015 consultation, Rwanda adopted its new Treat All policy, which changed ART eligibility to all HIV-positive persons regardless of CD4 count. Rwanda also adopted definitions of a “stable patient” who would be eligible for greater spacing between clinic visits – a strategy termed “differentiated care” that is designed to reduce transport, economic and logistic burdens of clinic visits on healthy patients, and thus promote greater long term retention in care.

It was recommended that for the first phase of the new service delivery model a stable patient would be defined as “a PLHIV >15 years old who has been on ART for 18 months with two viral load results of less than 20 copies/ml using Amplicor HIV-1 DNA test v1.5. and who is considered adherent (defined ≥90% adhering to taking their self-reported ART medication) under ART guidelines.” Under this initial definition, up to 85% of all PLHIV on ART would be defined as “stable.” The last viral load will be considered as recent if the results were reported at least after two months after blood collection for the test. All adult clients on second- and third-line treatments requesting to be part of the differentiated ART model, and fulfilling the eligibility criteria, are further evaluated and, if they show adequate adherence to treatment and viral suppression, are enrolled in the new ART model. Similarly, in accordance with other existing national guidelines, all members of key population groups can be included provided they meet the general eligibility criteria.

The following patients are currently considered non-eligible for the new model: all PLHIV clients on ART less than 18 months; patients on second and third line ART and not meeting adherence and viral load criteria; patients co-infected with NCDs such as diabetes, cancer, and heart disease in the intensive period of treatment until clinical symptoms are stabilized, pharmacy visit and laboratory visits will be coordinated with visits for their chronic conditions until patients is stabilized for that condition, that is patients hospitalized or in acute phase management; malnourished PLHIV (calculated body mass index/Height for Weight and excessive loss of weight below 10% of the normal value) in nutrition follow up services; all HIV positive clients who have TB and/or hepatitis (C and B) co-infections until after six months of ARVs treatment; and children under 15 years of age defined by the national HIV guidelines as paediatric patients.

Patients in this category will follow the standard ART guidelines: three monthly medical consultations, monthly medication pick up, monthly adherence counselling, and three monthly psychosocial support.

### Definition of the differentiated ART model

**Figure 2.**
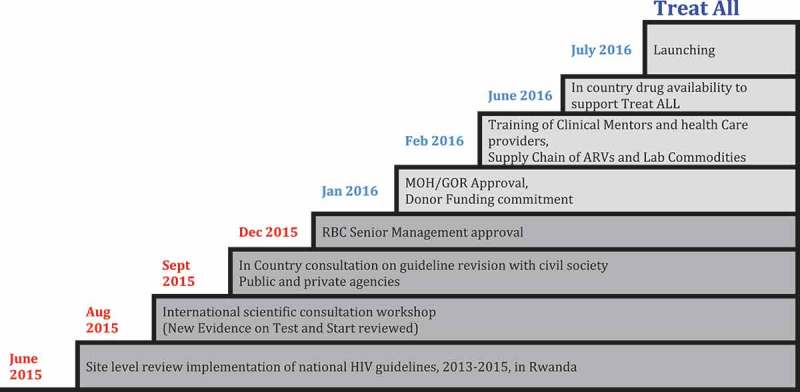
Implementation of “Treat All” in Rwanda.

With the introduction of the “Treat All” recommendations and the expanding availability of ART (Figure 2), it is anticipated that the majority of people will present to care earlier and require less intensive clinical care [12]. To reflect the different needs and preferences of different groups of PLHIV, and to reduce an unnecessary burden on the health system and multiple clinical visits for patients, the Rwandan national HIV programme adopted a differentiated model for ART service delivery.

The model aims to delink clinical visits from ART refills visits for stable patients by decreasing patient clinical visits to once every six months and pharmacy pick-up for medication visits (both ARVs and OIs prophylaxis) to once every three months [13,14]. Pharmacy visits will be spaced from one to three months, and clinical visits from three to six months for patients defined as stable [15,16]. Further, the length of adherence counselling sessions prior to initiating ART for patients who present well will be reduced from three to one to allow a rapid ART initiation for the newly identified patients – within 7 days and with the possibility of same-day start – consistent with recent evidence [17–19] (Table 1).

**Table 1.** “Standard of Care” vs. New Service Delivery Model

Group	Services	Standard of Care	New Service Delivery Model
Stable patients^a^	Clinical visits frequencies	3 months	6 months
	Medicine pick ups	1 month	3 months
	Counselling sessions (prior ART initiation)	3 sessions	1 session
	Peer support, CHWs	No	Yes
Specific populations: pregnant women, Key populations, Children <15 years, second, third lines, and TB or HB/CV co-infection	Clinical visits	3 months	3–6 months (Case by case)
	Medicine pick ups	1 month	1–3 months (Case by case)
	Counselling sessions (prior ART initiation)	3 sessions	2 Sessions

^a^Stable patients are defined as: >15 years old, on ART for 18 months with two viral load results of less than 20 copies/ml, and evidence of adherence to ART.

### Preparation for enrolment in the new model

Selection of stable patients for the new ART model is anticipated to take a month. High volume sites will enrol patients in cohorts (i.e. groups of patients attending the clinics at the same time) and these facilities will take a maximum of three months to enrol all patients in cohorts. The clinics can organize a reasonable number of cohorts given total number of patients (20–30) per cohort.

Health providers at health facility level will use patient registers and patient charts to classify patients receiving ART in different categories including: patients eligible for the new model; patients non-eligible for new model; and special considerations (pregnant and breastfeeding women, adolescents, patients with non-communicable diseases (NCDs), patients with other co-infections, and other considerations to be addressed on a case-by-case basis).

Health providers, supported by clinical mentors, will coordinate routine clinical consultations, planned medicine pickup with laboratory visits to reduce visit frequencies for patients. During the preparation phase, health providers will register stable patients (those eligible for the new spacing model) who have the same day visits and group them as a cohort. These patients/cohorts will be required to attend clinic on the same day to receive the same services.

Patients eligible for new ART delivery model are required to participate in one to two education and counselling sessions. During these sessions, health providers assess willingness to participate in the model, explain the benefits and risks related to the model, and summarize how the model works (how patients will be seen – either separately or in groups; coordination for different visits; community providers’ interventions; when patients move from one location to another, and so on). If eligible patients are pregnant women, adolescents, or patients with any chronic diseases that have a different consultation schedule, education sessions are adapted to a particular group, focusing on challenges for that specific group and how ART model will be aligned with the exiting conditions.

In parallel, support has been provided to community HIV services platforms to improve linkage and retention in treatment and adherence for all PLHIV including children born to key populations. At the community level, community healthcare workers and volunteers refer children to health facilities for HIV services. Facilities also work with community structures, including support groups and peer educators, for adherence support promotion, and retention in care.

#### Considerations for specific populations

Pregnant or breastfeeding mothers on ART will require different follow-up and health providers need to coordinate their clinical and pharmacy visits with ANC or PMTCT visits on a quarterly basis. A different frequency of clinic visits for pregnant women is used from the beginning of pregnancy until the end of the breastfeeding period. At the end of breastfeeding period, pregnant and breastfeeding mothers who meet all other eligibility criteria can then be enrolled in the new ART model. However, follow up of exposed and infected new born and younger children remains a special case requiring more regular contact with health services, both at the clinic and in the community.

Due to school routines and other competing priorities, adolescents at schools are scheduled for pharmacy and clinical visits every three months, timed with school break periods. All adolescents in school continue in this model until the end of their secondary education and at least 19 years of age. After this time they are transitioned to the spacing model as adult patients provided all other eligibility criteria are met. Adolescent out of school are be considered on a case-by-case basis based on virological, adherence and socioeconomic criteria.

### Engagement of implementing partners

Civil society and the private sector have been engaged to provide inputs through a civil society organization (CSO) consultation held in February 2016. This meeting engaged numerous community partners and their constituencies, including the NGO umbrella groups for health CSOs and PLHIV, as well as the CCM Secretariat, UNAIDS, and MOH/RBC. A second consultation was held during a mid-term review of the national ART programme in June 2016. During this process, civil society groups and implementing partners were encouraged to voice concerns on the new ART and service delivery model. Concerns were raised about implementation considerations: Increase of lost to follow up while contacts to clinics will be spaced- quality of household storage of drugs and associated losses or misuse.

In mid-2016, the RBC conducted a series of consultative meetings and workshops with health care providers, clinical mentors, district pharmacy directors, local leaders and community health workers. The objective of this consultation process was to seek support and feedback for the implementation of the new service differentiated model. During these consultations, experience sharing from the providers helped RBC to refine implementation steps including for example, the need to increase the storage space at the District Pharmacies.

#### Securing political and financial support

The recommendations from the technical working group were presented to the Minister of Health, Minister of State for Public health and Primary Health Care, and the Permanent Secretary for Health, for agreement in December 2015. Following this, a costed plan was presented to US President’s Emergency Plan for AIDS Relief (PEPFAR) to request US$4 million one-time additional central funding to cover the transition to three-month drug pick-ups nationally for stable patients and the related supply chain monitoring in order to increase the national buffer stock to support the rollout of the three-month drug pick-ups. PEPFAR approved US$3.67 millions of this request in June 2016 during PEPFAR’s Country Operational Plan (COP) review meeting.

Since 2005, the funding allocation for commodities in Rwanda is done by using a “common basket” mechanism; largely depending on external donor contributions. Under the Coordinated Procurement and Distribution System (CPDS) all actors in quantification, procurement and distribution from government, bilateral and multilateral partners meet regularly to estimate the needs and submit their report to Resource Management Committee (RMC) of the CPDS. The RMC requests financial contributions from each partner. In 2016, Of the total needs for ARVs, laboratory commodities and drugs for opportunistic infections prophylaxis and treatment; PEPFAR contributed 26 million USD (not including the central funding amount), the Global Fund contributed 19 million USD while The government of Rwanda has been investing in supporting infrastructure, running costs and personnel for HIV supply chain systems.

#### Forecasting drug supply

The national HIV Quantification Committee drafted a supply plan for all ARV drug needs in Rwanda in December 2015. The quantification exercise was supported with the use of *Quantimed*, a quantification software tool developed by USAID’s Strengthening Pharmaceutical Systems Project [20], took into account the implementation of “Treat All” in July 2016, and the required levels of buffer stock to transition to spaced clinic visits and dispensing for stable patients. It was estimated that 196,933 patients would be started on ART in 2017. In order to implement the three-month drug supply policy within the supply chain system as early as possible, a one-time procurement of additional products was determined to be necessary to top up all health facilities so they have the required stock to administer to patients. Based on procurement and supply chain systems, it was estimated to consider six months supply time for these additional quantities to be in place before the launch of the new guidelines.

Implementation of the three-month drug supply policy will occur as soon as the buffer stock was in place at facility level. In line with current practices within the national supply chain system, health facilities will calculate and order additional quantities necessary for subsequent orders. Additional shipments are needed for procurement to meet the demand on the national commodities supply due to the change in the service delivery model to three-month drug pick-ups. The cost of the entire ARV procurement needed to cover the transition was calculated at $US 3,677,180 USD. It was estimated that additional extra cost on other supply chain expenditures is minimal and could be managed within existing budget.

Ongoing monitoring of the supply chain on at least a quarterly basis will be put in place to adjust future shipments, monitor stock status, and avoid situations of expiry or stock out of medications. A full review of the implementation of the new policy and its effect on the distribution and storage system by the new supply chain mechanism is planned within the first quarter of rollout. Outputs from this review will inform future decisions regarding inventory control and distribution and procurement, as well as the national quantification of commodities, in order to realize cost savings and ensure commodity security. Based on all of these factors, the implementation of the three-month drug pick ups is, planned to start in December 2016.

#### Revision of ART guidelines

The revision of comprehensive HIV guidelines in Rwanda is carried out every two years based on most recent evidence available and priorities for the country and is a broadly consultative process involving experts from national technical working groups, implementing partners, bilateral and multilateral funding partners involved in HIV response in Rwanda, and external experts.

The revision and approval of the 2016 guidelines was concluded with the endorsement by the Ministry of Health. A nationwide training of health care providers was undertaken over a three-month period to ensure that at least two nurses in each of the 500 health centres in Rwanda were trained to implement the new guidelines. Trainings consisted of an overview on basics in HIV testing, treatment, and follow up, the benefits of the Treat All strategy, and evidence supporting differentiated service delivery and how best it can be implemented according to the country context. The trainings combined both expert presentations and groups discussions. At the end of each session, participants were encouraged to suggest improvements in the implementation plan. In addition, RBC has established clinical mentorship teams (one nurse and one doctor) who received advanced training in HIV management, located at each district hospital and supporting all health centres in the catchment area. These teams provide continuous education and skills transfer to the nurses responsible for managing ART. At the end of each training, participants received a written summary of the guidelines and the Ministry of Health issued an official letter to implement the new changes.

#### Dissemination and implementation

Each health centre was tasked to assess the number of stable patients and clinical services implementing partners will contact sites regularly to ensure that all sites are implementing the new service delivery model according to the national guidelines. Health centres are supported by clinical mentors to identify stable patients ahead of implementation. Implementation will be phased to allow for adaptation as necessary. Starting in October 2016, stable patients having an appointment will be asked to come back after six months and will be told to keep coming monthly for their drug pick ups only until the three-month drug pick ups are aligned with the patient’s new clinical visit schedule pending additional drugs for three months pick ups are expected to arrive in December 2016. These two appointments are conducted in different offices and drug pick up at pharmacy takes approximately five minutes while clinical visit in a consultation room is estimated to 20 min per patient. With an estimated average of ten consultations a day per health care provider, the spacing of clinical visits is expected to result in the following benefits: reduced burden on the clinics; increased time and quality of care for cases that need more clinical investigation; reduced frequency of travel for patients; and reduced likelihood of inadvertent disclosure of status as clients will spend less time in clinic services.

On 1 December 2016, patients with appointments for ARVs will receive 3 months supply if classified as stable and will be asked to come back one week before the end of their three-month stock. The transition to three-month drug pick-ups is expected to rollout over the course of three months so that at the end of the three-month period, beginning in December 2016, all stable patients will be receiving three months of drugs and will align one of every two three-month drug pick ups with one of the six-clinical visits (Table 2). During this period, special support will be needed to make adjustments in case issues arise.

**Table 2.** Roll out of three-month ARV pick-ups for stable patients

	Dec-16	Jan-17	Feb-17	Mar-17	Apr-17	May-17
Group A	3 months			3 months		
Group B	1 month	3 months			3 months	
Group C	1 month	1 month	3 months			3 months

Expected challenges include: stock outs (while additional bolus funding has been added to increase the buffer stock, adding an additional layer into the supply chain is a potential cause of stock out); loss to follow up (a potential negative effect of less frequent clinic contact); sharing of pills between patients; and issues of home storage of ARVs and confidentiality:

To mitigate these anticipated challenges, a quantification and distribution team has been established and tasked to meet every two weeks to monitor implementation and report regularly to the leadership of the RBC and MoH for real time changes and decisions making. The technical working group will continue to discuss the role of community workers in implementation of the new model in the framework of peer support to the network of people living with HIV.

Implementation of the new model will be adaptive so that on a quarterly basis, changes may be incorporated according to information and data received during implementation. For example: three-month pick ups may be better implemented in rural settings than in urban areas given high patients mobility in urban areas; if this is found to be the case, a two-month pick up may be proposed for such areas.

#### Monitoring and evaluation

The National HIV program with support from PEPFAR through CDC and WHO has established a technical group in strategic information to design how the new model will be evaluated on technical performance and financial reporting. Rwanda has a good M&E infrastructure for HIV with the TRACNet database that has been operating since 2004 and has recently been successfully integrated into Health Management Information System (HMIS). Since 2010 The TRACNet/HMIS has an interoperability system with Electronic Medical Record (EMR) and captures most of HIV individual patient data from testing to enrolment and follow up, covering 305 health facilities out of 500 existing in Rwanda. These systems will provide key data to track progress in the implementation of Treat All and outcomes of the new service delivery [21].

Enhanced indicators will be included such as number of patients that came for clinical visits and drug pick ups will be added in existing M&E systems. Financial indicators will be included based on visit and pick-up indicators, including case load of health workers and time spent to consult the patient.

## Conclusions

Based on the success of the new service delivery model, the Ministry of Health will review and seek to further strategically reduce costs to the national HIV program and to the patient by exploring and implementing adjustments to the service delivery model. For example, moving to 12-month clinical visits and 6-month prescription drug refills, and expanding the definition of “stable patient” to be more inclusive and reach more PLHIV. These and other modifications such as viral load results turn around time, adherence measurement to the service delivery model will seek to support the continued delivery of ART as part of the national HIV programme while maintaining quality.

## References

[1] UNAIDS Global AIDS Update. Geneva, Switzerland: UNAIDS; 2016.

[2] Elul B, Basinga P, Nuwagaba-Biribonwoha H, Saito S, Horowitz D, Nash D, et al High levels of adherence and viral suppression in a nationally representative sample of HIV-infected adults on antiretroviral therapy for 6, 12 and 18 months in Rwanda. Plos One. 2013;8(1):e53586. 23326462 10.1371/journal.pone.0053586 PMC3541229

[3] National Institute of Statistics of Rwanda (NISR), Demographic and Health Survey (DHS). Kigali, Rwanda. Available from http://www.statistics.gov.rw/publication/demographicandhealthsurveydhs-20142015-keyfindings

[4] Lu C, Chin B, Lewandowski JL, Basinga P, Hirschhorn LR, Hill K, et al Towards universal health coverage: an evaluation of Rwanda Mutuelles in its first eight years. Plos One. 2012;7(6):e39282. 22723985 10.1371/journal.pone.0039282 PMC3377670

[5] Binagwaho A, Farmer PE, Nsanzimana S, Karema C, Gasana M, De Dieu Ngirabega J, et al Rwanda 20 years on: investing in life. Lancet. 2014;384(9940):371–27. 24703831 10.1016/S0140-6736(14)60574-2 PMC4151975

[6] Organization WH Consolidated guidelines on the use of antiretroviral drugs for treating and preventing HIV infection: recommendations for a public health approach. 2nd ed. Geneva: WHO; 2016. 27466667

[7] Group TAS A trial of early antiretrovirals and isoniazid preventive therapy in Africa. N Engl J Med. 2015;373(9):808–22. 26193126 10.1056/NEJMoa1507198

[8] Group ISS, Lundgren JD, Babiker AG, Gordin F, Emery S, Grund B, Sharma S, et al Initiation of antiretroviral therapy in early asymptomatic HIV infection. N Engl J Med. 2015;373(9):795–807. 26192873 10.1056/NEJMoa1506816 PMC4569751

[9] Cohen MS, Chen YQ, McCauley M, Gamble T, Hosseinipour MC, Kumarasamy N, et al Antiretroviral therapy for the prevention of HIV-1 transmission. N Engl J Med. 2016;375(9):830–39. 27424812 10.1056/NEJMoa1600693 PMC5049503

[10] Rwanda Ministry of Health. National HIV annual report. 2015–2016, MOH. Kigali, Rwanda;2016.

[11] Anon 90–90–90. An ambitious treatment target to help end the AIDS epidemic. Geneva: UNAIDS; 2014.

[12] Mutimura E, Addison D, Anastos K, Hoover D, Dusingize JC, Karenzie B, et al Trends in and correlates of CD4+ cell count at antiretroviral therapy initiation after changes in national ART guidelines in Rwanda. AIDS. 2015;29(1):67–76. 25562492 10.1097/QAD.0000000000000520 PMC4487360

[13] Bemelmans M, Baert S, Goemaere E, Wilkinson L, Vandendyck M, Van Cutsem G, et al Community-supported models of care for people on HIV treatment in sub-Saharan Africa. Trop Med Int Health. 2014;19(8):968–77. 24889337 10.1111/tmi.12332

[14] Jain V, Byonanebye DM, Amanyire G, Kwarisiima D, Black D, Kabami J, et al Successful antiretroviral therapy delivery and retention in care among asymptomatic individuals with high CD4+ T-cell counts above 350 cells/mul in rural Uganda. AIDS. 2014;28(15):2241–49. 25022596 10.1097/QAD.0000000000000401 PMC4894849

[15] Babigumira JB, Castelnuovo B, Stergachis A, Kiragga A, Shaefer P, Lamorde M, et al Cost effectiveness of a pharmacy-only refill program in a large urban HIV/AIDS clinic in Uganda. Plos One. 2011;6(3):e18193. 21464895 10.1371/journal.pone.0018193 PMC3065481

[16] Nakiwogga-Muwanga A, Katabira E, Sempa J, Kambugu A, Nakibuuka-Lubwama E, Lamorde M, et al A pharmacy-only refill program at a large HIV clinic in Uganda: experience and satisfaction of patients. J Int Assoc Provid AIDS Care. 2014;13(3):264–68. 23744774 10.1177/2325957413488179

[17] Mutasa-Apollo T, Ford N, Wiens M, Socias ME, Negussie E, Wu P, et al. Effect of frequency of clinic visits and medication pick-up on antiretroviral treatment outcomes. J Int AIDS Soc. 2017;20(Suppl4), 21647. 10.7448/IAS.20.5.21647 PMC6192466 28770599

[18] Pilcher CD, Ospina-Norvell C, Dasgupta A, Jones D, Hartogensis W, Torres S, et al The effect of same-day observed initiation of antiretroviral therapy on HIV viral load and treatment outcomes in a US public health setting. J Acquir Immune Defic Syndr. 2017;74(1):44–51. 27434707 10.1097/QAI.0000000000001134 PMC5140684

[19] Rosen S, Maskew M, Fox MP, Nyoni C, Mongwenyana C, Malete G, et al Initiating antiretroviral therapy for HIV at a patient’s first clinic visit: the RapIT randomized controlled trial. Plos Med. 2016;13(5):e1002015. 27163694 10.1371/journal.pmed.1002015 PMC4862681

[20] Management Sciences for Health 2006. Quantimed: Pharmaceutical Quantification and Cost Estimation Tool. Arlington, Va. Retrieved from http://www.emtct-iatt.org/wpcontent/uploads/2013/02/Quantimed-Pharmaceutical-Quantification-and-CostEstimation-Tool

[21] Nsanzimana S, Remera E, Kanters S, Jamie IF, Nathan F, Jeanine C, et al Effect of baseline CD4 cell count at linkage to HIV care and at initiation of antiretroviral therapy on mortality in HIV-positive adult patients in Rwanda: a nationwide cohort study. Lancet HIV. 2015;2(9):e376–84. 26423551 10.1016/S2352-3018(15)00112-5

